# Targeting Tumor Stroma: Exploiting Apoptotic Priming

**DOI:** 10.18632/oncotarget.830

**Published:** 2012-12-31

**Authors:** Joachim C. Mertens, Gregory J. Gores

**Affiliations:** Division of Gastroenterology and Hepatology, Mayo Clinic, Rochester, MN; Division of Gastroenterology and Hepatology, University Hospital Zurich; Division of Gastroenterology and Hepatology, Mayo Clinic, Rochester, MN

**Difficulties in directly targeting tumor cells.** Specific therapeutic targeting of cancer cells has been the holy grail of oncological research for decades. However, effective cancer cell directed therapy has proven difficult to achieve mainly due to the enormous multitude of genetic aberrations in many types of cancer. Given the vast number of inter-individual genetic variations and biologic behaviours of malignant cells, each tumor patient can be considered to have his own private and individual tumor. Genetic diversity has even been described between cancer cells within a single tumor nodule [1]. This has led to an ever increasing diversification and complexity of cancer therapy under the grand theme of ‘individualized medicine’. Individualized medicine has resulted in modest oncologic success such as EGFR and ALK targeted therapy in non-small cell lung cancer [2]. However, therapeutic progress has been slow despite considerable progress in DNA sequencing of cancers.

**Tumor stroma as important factor in tumor biology – the tumor ‘ecosystem’.** Malignant cells exist within a complex ‘ecosystem’ [3] consisting of a variety of cell types. Stromal cells of the tumor microenvironment have been shown to play a crucial role in tumor development, growth and metastasis. An important cell population aside from the malignant cells themselves are activated, α-smooth muscle actin positive fibroblasts of the tumor microenvironment. These cells, also called cancer associated fibroblasts or CAF provide growth factors, modify and secrete extracellular matrix and facilitate cancer cell survival and metastatic mobility [4]. Of the multitude of interactions between tumor and stromal cells, the secretion of PDGF and the extracellular matrix components tenascin C and periostin by CAF have been demonstrated to promote tumor growth and metastasis [5]. Also the number of CAF in the tumor microenvironment of cancer patients is inversely correlated with patient survival. Thus CAF are a potential therapeutic target in human cancers.

**Concept of apoptotic priming.** Activated cell states have been described to result in an increased cellular sensitivity towards apoptotic stimuli. This is known for T-cells, which become susceptible to apoptotic cell death upon activation [6] as well as activated hepatic stellate cells in acute liver injury. The phenomenon of increased apoptotic sensitivity has been termed ‘mitochondrial priming’, as apoptosis in these cells occurs though the mitochondrial pathway. Mitochondrial priming can be determined by assessing the mitochondrial release of apoptotgenic factors upon incubation with different pro-apoptotic BH-3 peptides [8]. These peptides mimic the binding activities of BH3-only proteins, pro-apoptotic members of the Bcl-2 family of proteins, which trigger the mitochondrial apoptotic pathway. Still the underlying mechanisms of ‘mitochondrial priming’ are not well understood. Moreover tumor stroma cells have not been previously examined with respect to their potential mitochondrial priming during the process of their cellular activation.

**Activated CAF display apoptotic priming.** Given the above information, we sought to determine if CAF can be selectively targeted by pro-apoptotic therapy. We observed, that the paradigm of activation induced mitochondrial priming is also true for the activated CAF in the tumor microenvironment [7]. We profiled Bcl-2 proteins in CAF and quiescent stromal cells and observed an increased expression of Bax, a potent multidomain pro-apoptotic protein of the Bcl-2 family. We next employed the pro-apoptotic BH-3 mimetic navitoclax (ABT-263) and observed a strong induction of apoptosis in the CAF *in vitro*, while quiescent fibroblasts were completely unresponsive and malignant cell lines showed minimal sensitivity. Malignant cholangiocarcinoma cells are likely resistant to navitoclax as they overexpress Mcl-1, a potent anti-apoptotic Bcl-2 protein which does not bind navitoclax. The upregulation of Bax helps to prime the mitochondria, sensitizing CAF to navitoclax cytotoxicity. Interestingly we were able to generate a similar phenotype in previously quiescent fibroblasts by pretreating these cells with TGF-β, as TGF-β effectively activates and sensitizes them to navitoclax-induced apoptosis. Taken together, these observations suggest targeting tumor stroma cells with pro-apoptotic compounds represents a novel therapeutic approach in cancer.

**Figure 1:**
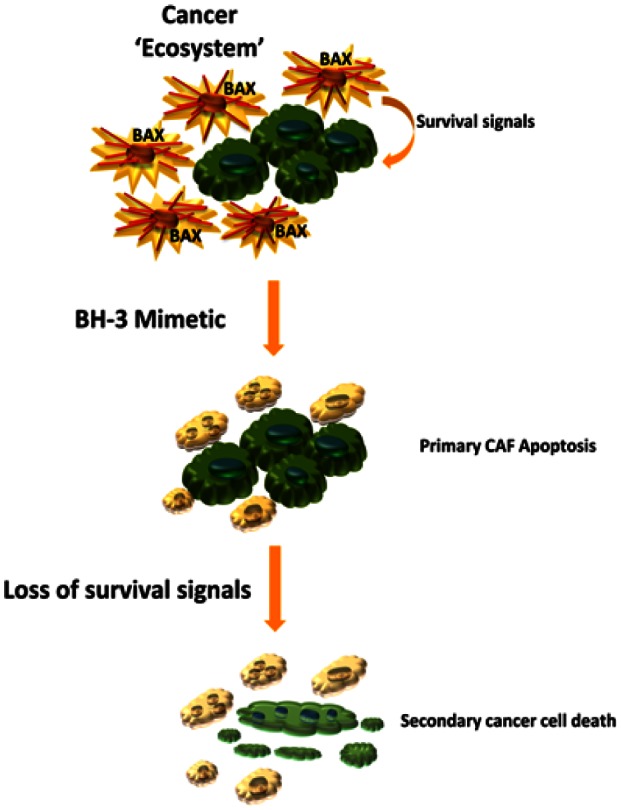
Therapeutic deletion of CAF for the treatment of highly desmoplastic human cancers Activated cancer associated fibroblasts, CAF, (spiculated orange cells) display mitochondrial priming for apoptotic cell death – presumably due to upregulation of the pro-apoptotic Bcl-2 protein Bax. As such these cells can be targeted with a BH3 mimetic, inducing CAF apoptosis and subsequently depleting the cancer cells (green cells) of important stroma-derived survival signals. The cancer cells then undergo secondary apoptosis.

**Targeting tumor stroma with BH3 mimetics.** To test the hypothesis of mitochondrial priming and sensitivity to pro-apoptotic therapeutics *in vivo* we employed a highly aggressive orthotopic, syngeneic animal model of cholangiocarcinoma. Indeed navitoclax did induce Bax activation, release of pro-apoptotic factors and apoptotic cell death primarily in activated CAF. Targeted deletion of CAF results in a secondary reduction of tumor size, metastasis and an improved animal survival (Fig. 1).

**Perspectives of combined therapeutic approaches.** As effective and specific therapies targeting only the malignant cells are still a distant prospect in many cancers, combination cancer therapies, targeting CAF and the cancer cells may be the more promising and realistic therapeutic objective. In this multimodal approach, the tumor stroma offers a more uniform, less genetically heterogeneous target that should be a focus of future therapeutic developments. Exploiting the apoptotic priming of tumor stromal cells combined with other tumor cell targeted therapies could be an elegant approach for the treatment of desmoplastic cancers.
